# Modulating Surface Properties and Osteoblast Responses in Bone Regeneration via Positive and Negative Charges during Electrospinning of Poly(l‑lactide-*co*-ε-caprolactone) (PLCL) Scaffolds

**DOI:** 10.1021/acsbiomaterials.5c01568

**Published:** 2025-11-27

**Authors:** Katarzyna Marszalik, Martyna Polak, Krzysztof Berniak, Joanna Knapczyk-Korczak, Piotr K. Szewczyk, Mateusz M. Marzec, Urszula Stachewicz

**Affiliations:** † Faculty of Metals Engineering and Industrial Computer Science, AGH University of Krakow, Al. A. Mickiewicza 30, Krakow 30-059, Poland; ‡ Academic Centre for Materials and Nanotechnology, AGH University of Krakow, Al. A. Mickiewicza 30, Krakow 30-059, Poland

**Keywords:** electrospinning, fibers, voltage polarity, PLCL, copolymers, cell−material interactions, collagen formation

## Abstract

The global demand for faster and more effective bone regeneration calls for biomimetic scaffolds that actively guide cell behavior beyond providing structural support. Electrospinning offers unique opportunities to tailor scaffold properties, yet the influence of positive and negative voltage polarities during fabrication on cell–material interactions remains largely unexplored. Here, we investigate poly­(l-lactide-*co*-ε-caprolactone) (PLCL) scaffolds, a statistical copolymer combining strength and elasticity, produced under positive (PLCL+) and negative (PLCL−) polarity. Both scaffold types display comparable morphologies and bulk chemistry. However, X-ray photoelectron spectroscopy reveals charge dependent surface chemistry, with PLCL– enriched in O=C and O–C groups. Zeta potential results highlight pronounced voltage polarity effects under aqueous conditions at pH 7.5, showing −29.19 mV for PLCL+ and −34.77 mV for PLCL–. Biologically, both scaffolds support rapid osteoblast attachment, with robust filopodia and collagen type I deposition by day 14. Strikingly, PLCL+ scaffolds promote deeper cellular infiltration and broader cytoskeletal distribution, whereas PLCL– scaffolds enhance proliferation, but with a flatter cell morphology. These findings reveal that subtle, charge-driven surface chemical differences in random copolymer scaffolds profoundly modulate osteoblast behavior. This work identifies electrospinning voltage polarity as a powerful yet underutilized design parameter for engineering next-generation scaffolds for bone tissue regeneration.

## Graphical abstract

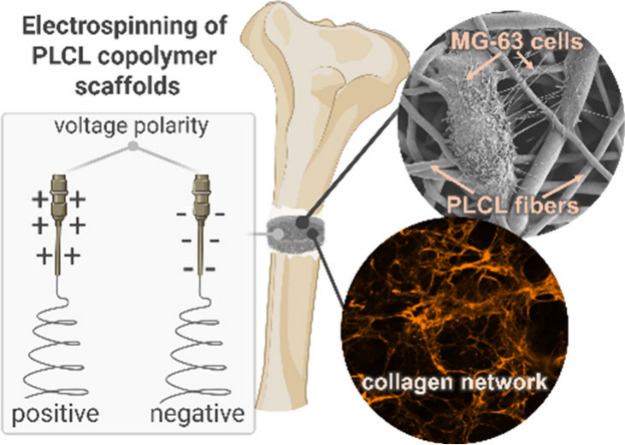

## 1 Introduction

With an aging population and an increasing incidence of bone injuries and diseases, the global demand for effective bone regeneration strategies continues to rise. Each year, millions of patients worldwide undergo orthopedic surgeries, often requiring bone grafts or implants, with a growing emphasis on accelerating healing and functional recovery.^1,2^ Tissue engineering offers a promising alternative to traditional bone grafts by developing scaffolds that not only provide structural support but also stimulate biological activity.^3–5^

A critical feature of successful scaffolds is their ability to promote key cellular behaviors, including adhesion, proliferation, migration, and extracellular matrix (ECM) formation.^6–12^ Collagen, particularly type I collagen, is essential to bone matrix development, providing both the structural framework and mechanical resilience required for mineralization and functional recovery.^13^ Among fabrication methods, electrospinning is widely recognized for its ability to create nanofibrous scaffolds that mimic the architecture of natural ECM.^9,14,15^ These scaffolds exhibit a high surface-area-to-volume ratio, interconnected porosity, and tunable architecture, all of which support cell infiltration and tissue formation.^16–18^ Importantly, electrospinning allows precise control over fiber morphology and surface chemistry by tuning solution properties (e.g., viscosity, concentration),^19–22^ process parameters (e.g., voltage, flow rate),^23–26^ and environmental conditions (e.g., temperature, humidity).^27–29^ Furthermore, studies have shown that voltage polarity during electrospinning influences the surface charge and potential of fibers, key factors governing cell–material interactions.^7,30–34^ While much focus has been given to material composition and topography, surface potential has emerged as a critical, yet underexplored, cue that affects early cellular responses and ECM development. Our group previously demonstrated that electrospun poly­(ε-caprolactone) (PCL) scaffolds produced under different voltage polarities exhibited distinct surface potentials without altering fiber morphology.^31,33^ Specifically, fibers electrospun using negative voltage polarity (PCL−) displayed higher surface potential and promoted enhanced osteoblast adhesion, filopodia formation, and collagen deposition compared to PCL+. Similarly, for poly­(l-lactide) (PLLA), we found that positive voltage polarity yielded significantly higher surface potential and enhanced early cell adhesion, though long-term effects on proliferation and ECM formation were less pronounced.^30^ Despite these insights, the influence of voltage polarity on copolymer scaffolds, particularly poly­(l-lactide-*co*-ε-caprolactone) (PLCL), remains unexplored. PLCL combines the elasticity of PCL with the stiffness of PLLA, offering tunable degradation and mechanical properties favorable for both soft and hard tissue engineering.^35,36^ Given its widespread use, understanding how to modulate its surface properties via electrospinning could unlock further improvements in scaffold performance. Therefore, this study addresses this critical knowledge gap by investigating how electrospinning voltage polarity influences the surface characteristics and biological behavior of PLCL copolymer scaffolds. We evaluated changes in surface potential, chemistry, and cellular responses, including adhesion, proliferation, cell replication, and early collagen expression, without altering the bulk polymer composition. We also analyzed the cell–scaffold interfaces, with a focus on cell spreading and filopodia-mediated attachment, to better understand how surface potential shapes early tissue responses. The concept of the study is illustrated in Figure 1. We demonstrate that voltage polarity enables tunable control of the surface potential, governed by the polymer architecture and its ability to undergo molecular reorientation. Importantly, PLCL scaffolds supported robust early expression of type I collagen, confirming their suitability for bone regeneration and underscoring the importance of further studies of charge modulation in complex polymer systems.

**Figure 1:**
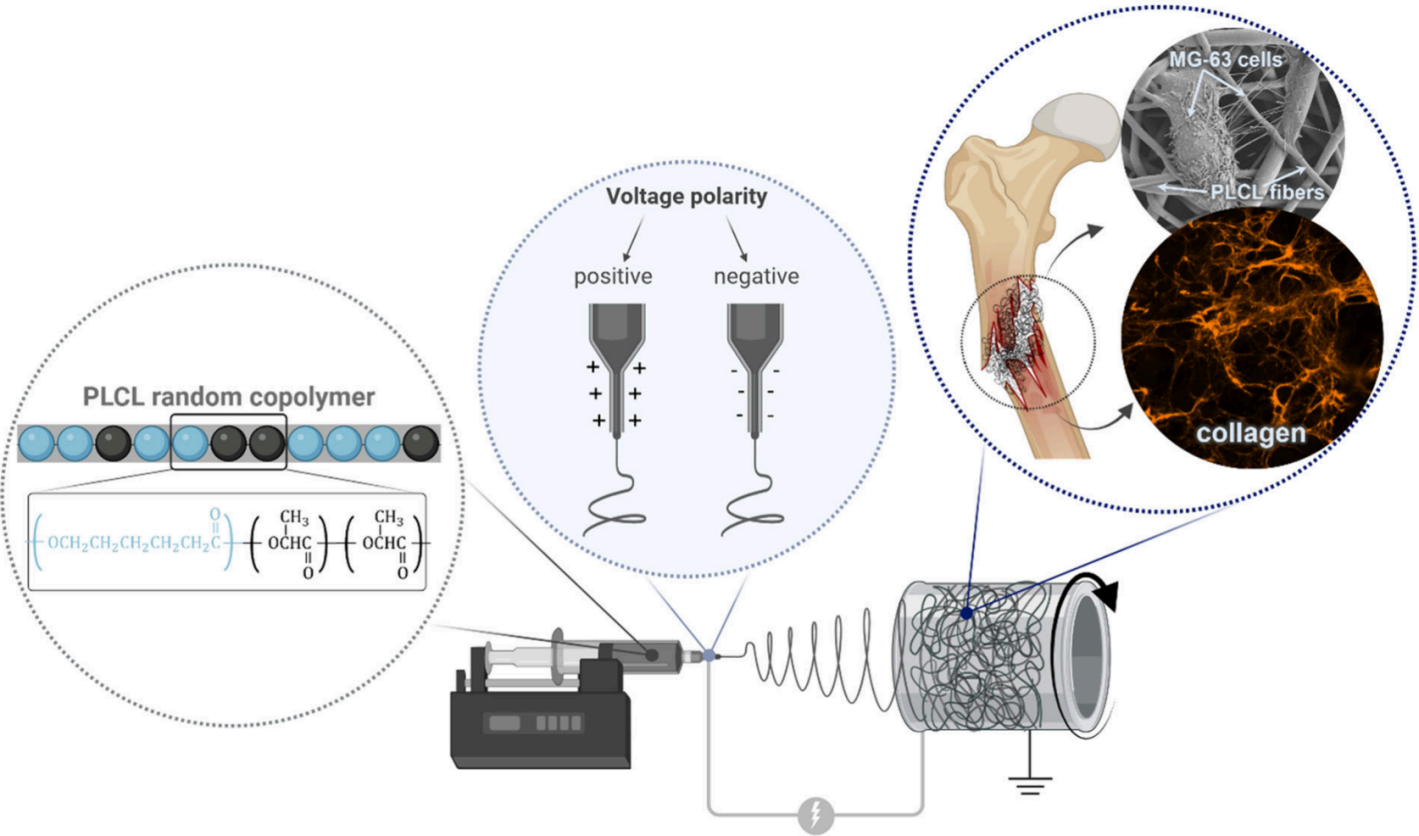
Conceptual scheme of the study on the effect of positive and negative voltage polarity on the surface properties of PLCL copolymer scaffolds and corresponding cell responses, indicating their potential for use in bone tissue regeneration.

## 2 Results and Discussion

### 2.1 Scaffold’s Morphological, Chemical, and Mechanical Characterization

The change of a single electrospinning parameter can significantly affect the properties of the resulting fibers without any additional postprocessing.^37^ Here, we investigate how applying positive or negative voltage polarity to the nozzle influences the properties of electrospun PLCL fibers. In general, alternating the polarity between positive and negative leads to the accumulation of opposite charges on the surface of the polymer jet.^25^ As a result, the orientation of molecular dipoles and polymer chains within the jet differs depending on the applied voltage polarity, thereby influencing the morphology, chemical composition, mechanical performance, and surface properties of the fibers. The detailed mechanism driving these polarity-dependent molecular rearrangements that occur under positive and negative voltages is discussed in section 2.2 (Surface Properties of Scaffolds).

In this study, two types of electrospun fibrous scaffolds were successfully fabricated by using the same PLCL copolymer solution under opposite voltage polarities during electrospinning. A schematic overview of the fabrication setup and macroscopic images of the resulting PLCL scaffolds are presented in Figure 2a. The selection of the solvent system for electrospinning of PLCL fibrous scaffolds was based on a comprehensive literature review. The morphology and stability of PLCL fibers depend on the solvent system and key electrospinning parameters, including polymer concentration, humidity, and applied voltage.^38–40^ Chloroform provides excellent solubility of PLCL.^36,39,41–43^ Previous studies have demonstrated that the incorporation of methanol into solutions results in enhanced solution conductivity, reduced fiber diameter, and improved jet stability during processing.^44^ A small amount of acetic acid promotes a more homogeneous charge distribution and reduces the bead formation effects previously reported for similar polyester systems.^45^ Therefore, an 8:1:1 (v/v/v) solvent mixture of chloroform, methanol, and acetic acid was chosen to prepare a 10 wt % PLCL solution. Complete polymer dissolution and a stable process were achieved using the selected solvent mixture. The influence of different voltage polarities on the morphology of the fibrous scaffolds was investigated using scanning electron microscopy (SEM). Representative SEM micrographs revealed smooth, uniform PLCL+ and PLCL– fiber surfaces in the absence of beads and pores (Figure 2b,c). The average fiber diameters were 1.96 ± 0.43 and 1.97 ± 0.48 μm for PLCL+ and PLCL–, respectively (Figure 2d). Morphological analysis showed that the electrospun PLCL scaffolds exhibited fiber diameters comparable to those reported for PLLA and PCL scaffolds fabricated under varying voltage polarities. The average fiber diameter was 2.2 ± 0.7 μm for PLLA+, 2.4 ± 0.9 μm for PLLA–, 3.9 ± 1.6 μm for PCL+, and 3.7 ± 1.1 μm for PCL–.^30,31^ These results suggest that despite slight variations in fiber diameter distribution among the materials, the overall morphology is sufficiently comparable to allow consistent interpretation of our results across materials. The fibers and pore sizes influence each other and affect cell infiltration.^46^ In the 2D analysis, SEM micrographs of PLCL fibrous scaffolds were used to quantify the pore area and the percentage of porous surface area on the analyzed region. The average pore size was similar for both samples, with values of 10.80 ± 8.21 μm^2^ for PLCL+ and 9.60 ± 7.20 μm^2^ for PLCL–. Similarly, the percentage of the porous area was 19.26% for PLCL+ and 11.39% for PLCL–. Detailed pore size distribution profiles are presented in Figure S1a in the Supporting Information.

**Figure 2:**
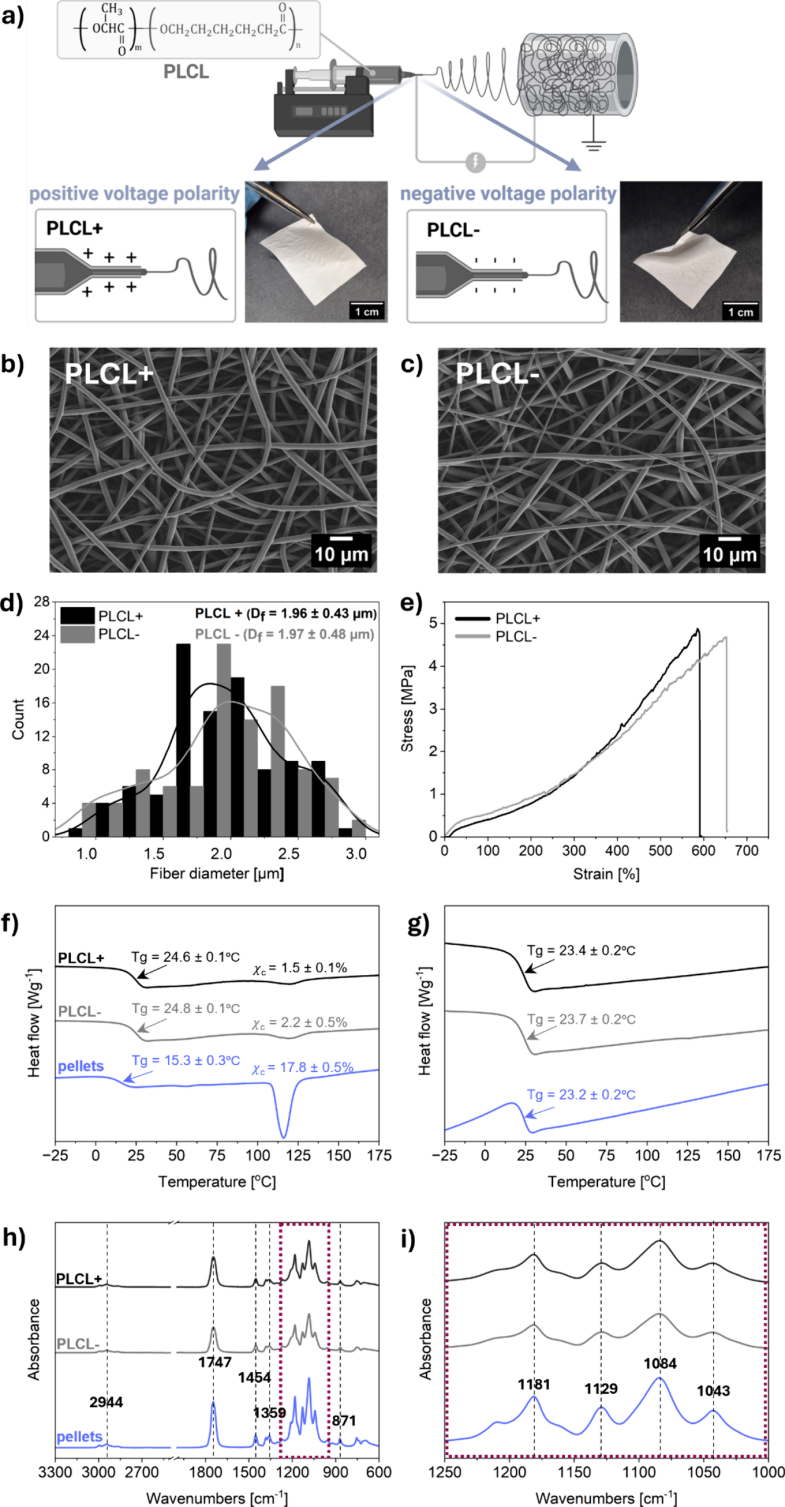
(a) Structural formula of the PLCL copolymer^57^ and schematic illustration of the electrospinning setup with positive and negative voltage polarities, showing the distribution of corresponding charges on the cone-jet of the polymer solution, along with photographs of the electrospun PLCL+ and PLCL– fibrous scaffolds. SEM micrographs of (b) PLCL+ and (c) PLCL– scaffolds, (d) histogram of fiber diameter distribution with Kernel density smoothing, (e) stress–strain curves obtained after tensile testing of PLCL+ and PLCL– scaffolds, (f) results of 1st DSC scan, (g) results of 2nd DSC scan, (h) full FTIR spectra, and (i) FTIR spectra focused on the 1181, 1129, 1084, and 1043 cm^–1^ peaks, showing a zoomed-in region marked with dashed red lines in (h).

The wetting properties were evaluated through the measurement of water contact angles, which resulted in 121 ± 1° for PLCL+ and 127 ± 2° for PLCL– (Figure S1b in the Supporting Information). This measurement indicated the hydrophobic nature of the PLCL fibrous scaffolds. Similarly, fibrous scaffolds produced from PLLA and PCL using different voltage polarities have also been reported to exhibit hydrophobic surface properties.^30,31^ To determine the effect of the voltage polarity on the mechanical performance of PLCL random fibers during electrospinning, uniaxial tensile testing was performed on PLCL+ and PLCL– samples. The evaluated mechanical parameters include tensile strength (σ_max_), strain at maximum stress (ε_max_), strain at failure (ε_f_), and toughness (*W*) as summarized in Table 1. Figure 2e shows the representative curves chosen from 3 measurements per sample, whereas all the curves are presented in Figure S2 in the Supporting Information. Both types of PLCL scaffolds exhibited comparable mechanical performance. The average σ_max_ values were similar for both samples, PLCL+ (4.74 ± 0.19 MPa) and PLCL– (4.42 ± 0.23 MPa). ε_f_ values were slightly higher in the PLCL– sample (633.86% ± 21.10%) compared to the PLCL+ sample (595.26% ± 17.44%), but the difference was not statistically significant, indicating comparable extensibility. The toughness values, which represent the energy absorbed before failure, were similar for both samples 10.84 ± 1.01 MJ·m^–3^ for PLCL+ and 10.91 ± 1.93 MJ·m^–3^ for PLCL–, indicating consistent energy dissipation during deformation. We noted that the mechanical properties of the PLCL scaffolds were not significantly affected by the variation in voltage polarity applied during the electrospinning. The Young’s modulus of the randomly oriented electrospun fiber mats was not determined due to fiber slippage and the nonlinear deformation behavior observed during tensile testing. As indicated in previous studies, the values obtained for the Young’s modulus of random electrospun fiber mats can be misleading.^47–49^

**Table 1:** Tensile Test Results for PLCL+ and PLCL– Scaffolds^a^

Sample	σ_max_ [MPa]	ε_max_ [%]	ε_f_ [%]	*W* [MJ·m^–3^]
PLCL+	4.74 ± 0.19	587.22 ± 15.76	595.26 ± 17.44	10.84 ± 1.01
PLCL–	4.42 ± 0.23	613.35 ± 35.06	633.86 ± 21.10	10.91 ± 1.93

a Characteristic values of tensile strength (σ_max_), strain at maximum stress (ε_max_), strain at failure (ε_f_), and toughness (*W*). Errors are based on standard deviation, with *N* = 3, where *N* is the tensile test of one mat sample.

Differential scanning calorimetry (DSC) measurements revealed no significant differences in glass transition temperature (*T*_g_) or melting temperature (*T*_m_) between the PLCL+ and PLCL–, see Table 2. No difference in *T*_g_ was observed between the first and second heating scans, indicating that the positive or negative voltage polarity had no measurable effect on the thermal properties of the polymer fibers (Figure 2f,g). A marginal increase in crystallinity (χ_c_) was observed for the PLCL– sample (2.2 ± 0.5%) compared to that of the PLCL+ sample (1.5 ± 0.1%). Both samples exhibited substantially lower crystallinity than the PLCL pellets (17.8 ± 0.5%). The reduced crystallinity of PLCL fibers in comparison to pellets is attributed to the electrospinning parameters. During the solidification and stretching of fibers, polymer chains have limited time to orient themselves into a crystalline structure prior to solidification.^50–52^ Our results suggest that the electrical charges applied during fiber formation from the PLCL copolymer do not significantly impact the crystalline structure and thermal behavior of the scaffolds.

**Table 2:** DSC Results for PLCL+ and PLCL– Scaffolds^a^

	1st DSC scan				2nd DSC scan
Sample	*T*_g_ [°C]	*T*_m_ [°C]	Δ*H*_m_ [J·g^–1^]	χ_c_ [%]	*T*_g_ [°C]
PLCL+	24.6 ± 0.1	120.1 ± 0.5	1.1 ± 0.04	1.5 ± 0.1	23.4 ± 0.2
PLCL–	24.8 ± 0.1	118.8 ± 3.7	1.6 ± 0.40	2.2 ± 0.5	23.7 ± 0.2
pellets	15.3 ± 0.3	115.2 ± 1.1	13.2 ± 0.40	17.8 ± 0.5	23.2 ± 0.2

a Characteristic values of glass transition temperature (*T*_g_), melting temperature (*T*_m_), enthalpy of melting crystallization (Δ*H*_m_), and crystallinity (χ_c_).

Fourier transform infrared spectroscopy (FTIR) analysis confirmed that the PLCL+ and PLCL– samples have similar bulk chemical compositions compared to the PLCL pellets, see Figure 2h,i and Table S1 in the Supporting Information). The spectrum of PLCL showed a characteristic carbonyl (C=O) stretching at peak at 1747 cm^–1^, corresponding to the ester group.^53,54^ The peak observed at 2944 cm^–1^ was attributed to C–H stretching vibrations,^55^ while the peaks at 1454 and 1359 cm^–1^ were associated with −CH_3_ bending.^53^ The C–O stretching at peaks in the 1000–1300 cm^–1^ region confirmed the presence of ester groups, with absorption peaks at 1181, 1129, 1084, and 1043 cm^–1^.^54^ Additionally, a weaker peak at 871 cm^–1^ indicated the presence of a C–COO stretching bond.^56^

### 2.2 Surface Properties of Scaffolds

Electrospinning requires applying either a positive or negative electrical polarity to the nozzle, which significantly affects the surface properties of polymer fibers.^58^ During the initial stage of the process, charges of the same sign as the applied voltage polarity accumulate on the jet surface.^25^ Applying an electrical potential to the liquid interface of a polymer solution affects the surface chemistry of the electrospun fibers. When the nozzle is positively charged, the jet moving toward the grounded collector carries positive charges on its surface. Furthermore, part of the jet’s bulk charges that have not yet migrated to the surface flow within the jet, driven by the electric field. The situation is reversed when a negative voltage polarity is applied. Therefore, the applied voltage polarity can easily cause the reorientation of polymer chains in a polymer solution jet. During electrospinning, the polymer chains in the solution are highly responsive to the applied electric field, which can induce molecular reorientation. The rotation of dipoles under the electric field corresponds to the polarization within the polymer jet. As a result, the electronegative segments of the polymer chains can be attracted to or repelled by the surface charges, leading to their reorientation toward or away from the liquid–air interface. The jet then continues to elongate toward the collector, where it solidifies, preserving the specific dipole and chain conformations established before fiber deposition on the collector. Figure S3 in the Supporting Information demonstrates the schematic illustration of polymer chain reorientation in electrospinning after applying positive or negative electrical polarity. The hypothesis of reoriented polymer chains during the electrospinning of fibers from the following polymers has been confirmed by a few studies using polyethylene oxide (PEO),^59^ polymethylmethacrylate (PMMA),^58,60^ polycarbonate (PC),^61^ and polyamide 6 (PA6),^32^ as well as PLLA^30^ and PCL,^31^ which are used to synthesize the PLCL copolymer. This molecular reorientation mechanism provides a basis for understanding the observed polarity-dependent differences in surface chemistry and cell response discussed below.

The chemical structure of the PLCL copolymer includes polar functional groups such as C=O and C–O bonds (Figure 3a). These bonds exhibit a significant difference in electronegativity between carbon and oxygen, generating a dipole moment in which oxygen carries a partial negative charge and carbon a partial positive charge.^62,63^ During electrospinning, the application of an external electric field, particularly under different voltage polarities, can induce reorientation of these dipolar groups toward or away from the fiber surface.^25^ The angle-resolved X-ray photoelectron spectroscopy (ARXPS) analysis was used to evaluate the potential reorientation of polymer chains toward the fiber surface. Surface concentrations of chemical bonds obtained from fitting ARXPS data for PLCL+ and PLCL– are listed in Table S2 in the Supporting Information. Although no structural or mechanical differences were observed through FTIR, DSC, and in a tensile test; ARXPS revealed distinct variations in the surface chemical properties of the PLCL+ and PLCL– fibers. Our study focused on verifying the presence of double- and single-bonded oxygen content. The O 1s spectra were fitted with two components, the first line centered at 532.2 eV, which points out the existence of O=C type bonds, and a second line centered at 533.6 eV indicating the presence of O–C type bonds, see Figure 3b.^64^ The chemical composition of the PLCL+ and PLCL– fibers was examined by ARXPS at a 45° angle, which corresponded to a measurement depth of 5.7 nm. The ARXPS results revealed differences in the chemical compositions of the PLCL+ and PLCL– fibers. For the PLCL+ fibers, the concentration of O=C was lower at 13.6%, whereas for the PLCL– fibers it was higher at 15.4%. A similar observation was made with regard to the O–C bond. For PLCL+, the amount was 16.6%, whereas for PLCL–, it was higher at 17.8% (Figure 3c). The ARXPS results suggest that the reorientation of oxygen in the PLCL molecules occurs due to a change in polarity, which is confirmed by the different O 1s intensity of the PLCL+ and PLCL– fibers. Although the compositional differences between the samples electrospun with opposite voltage polarities are relatively small (1–2%), previous studies of PCL and PLLA have shown that even minor variations can significantly impact the surface potential of fibers.^30,33^

**Figure 3:**
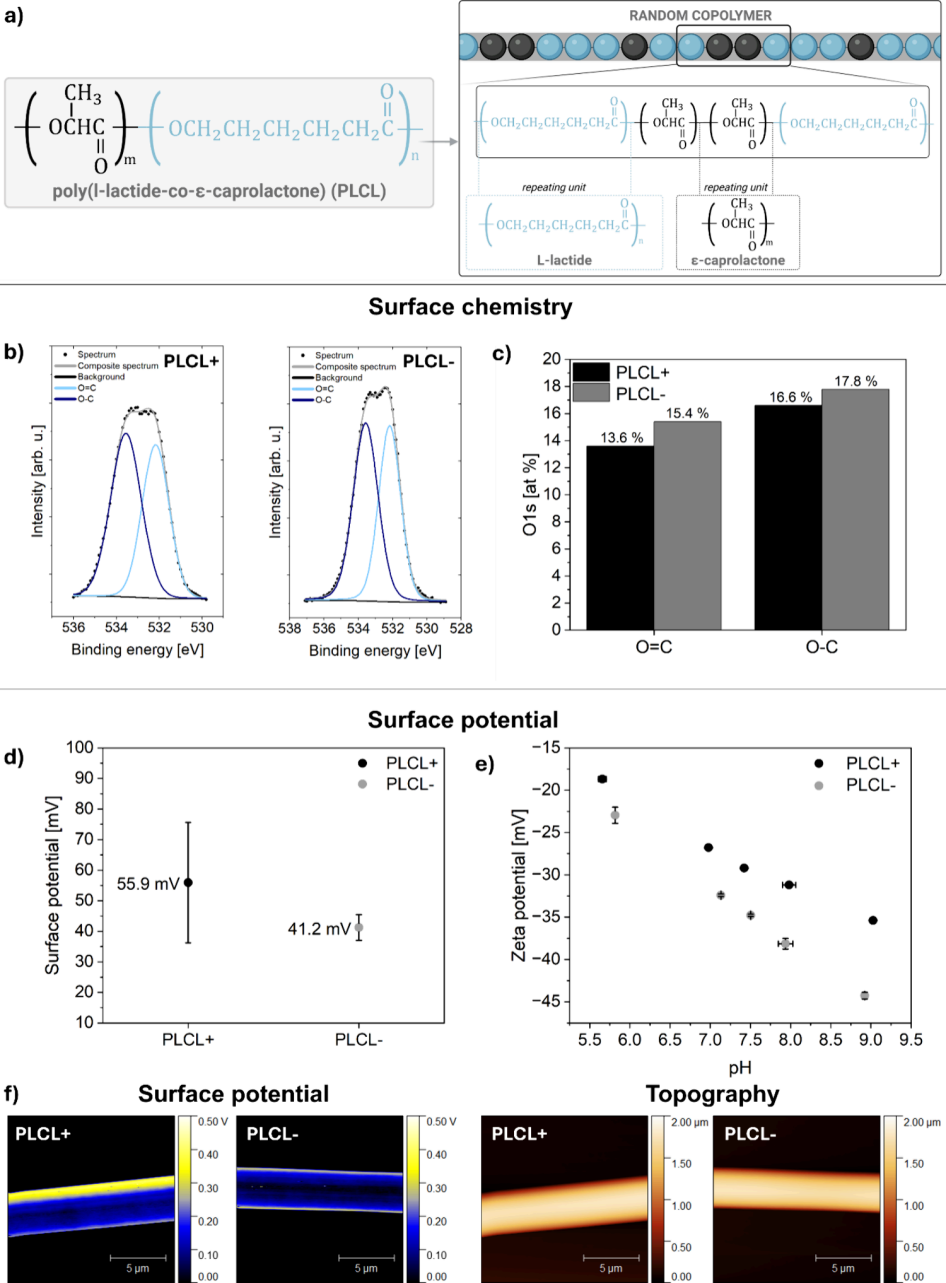
(a) Molecular structure of PLCL random copolymer, showing the chemical structure of the repeating units and a schematic representation of the copolymer chain architecture with randomly distributed l-lactide and ε-caprolactone segments; (b) ARXPS spectra of the O 1s region for PLCL+ and PLCL– taken at 45° (information depth ≈ 5.7 nm); for all spectra, two lines were fitted corresponding to oxygen in the O–C (533.5 eV, navy blue) and O=C (532.2 eV, blue) bonds; (c) atomic composition in terms of single and double bonded oxygen (%) of the surface of electrospun samples determined by fitting ARXPS data, taken at 45° (information depth ≈ 5.7 nm); (d) surface potential of fibers measured via KPFM, (e) zeta potential results in the pH range of approximately 5.5–9, and (f) KPFM surface potential maps with corresponding AFM topography images of individual electrospun PLCL+ and PLCL– fibers.

Electrospinning fibers with positive or negative polarity affects not only the surface chemistry of the fibers but also their surface potential. Previous studies have shown that modifying the surface chemistry of electrospun fibers using different voltage polarities results in changes to the surface potential, which can be examined using Kelvin probe force microscopy (KPFM) and zeta potential measurements.^25,30,32,33,60^ In contrast to previously reported results for homopolymers such as PLLA and PCL, where electrospinning under different voltage polarities induced variations in surface potential, electrospun fibers from the PLCL copolymer exhibited only a minor shift in surface potential values. For PLCL+, the surface potential was 55.93 ± 19.68 mV, while for PLCL–, it was 41.23 ± 4.22 mV, see Figure 3d. KPFM maps illustrating the fiber topography and the corresponding surface potential of PLCL fibers are shown in Figure 3f. In contrast, KPFM measurements revealed that PLLA+ fibers exhibited an average surface potential shift of approximately 295 mV compared to PLLA– fibers.^30^ It was previously reported that electrospinning PCL under different voltage polarities led to differences in surface potential, with a shift of approximately 20 mV.^31^ The surface potential was estimated to be 554.7 ± 12.8 and 574.4 ± 11.2 mV for the PCL+ and the PCL– fibers, respectively. These results indicate that the random structure of PLCL limits its ability to respond to surface modifications, making it difficult to adjust its surface properties by changing the voltage polarity during electrospinning. PLCL can be synthesized as a random copolymer through the simultaneous or sequential addition of l-lactide (LA) and ε-caprolactone (CL) or as a block copolymer in which distinct PCL and PLLA blocks are formed in a controlled sequence.^65,66^ The resulting monomer distribution directly impacts the copolymer’s physicochemical and mechanical properties. An increased LA ratio enhances tensile strength and stiffness, while a higher CL content improves elasticity and flexibility.^36,66^ The random copolymer architecture of PLCL results in a statistical distribution of polar functional groups like C=O and C–O along the polymer chain (Figure 3a).^67^ This irregular arrangement can prevent consistent dipole reorientation in response to an electric field during electrospinning. Consequently, attempts to modify surface chemistry by altering the voltage polarity are largely ineffective because local dipole cancellation minimizes net changes in surface potential. Thus, the random distribution of polar groups in PLCL limits their ability to align under an electric field, resulting in minor changes to the surface potential of the individual fibers. Future studies comparing random and block PLCL architectures under the same electrospinning conditions are necessary to understand how the monomer sequence affects surface responsiveness. More ordered copolymer structures should enable greater modulation of surface properties.

Zeta potential is a measurement of the surface charge of a material in its physiological environment.^30^ It is pivotal in understanding the behavior of the functional groups from the surface of the fibers in contact with acid or base fluids, which can be indicative of the pH of biological fluids. This critical information facilitates a comprehensive understanding of the adhesion of proteins influenced by this surface potential and the subsequent process of tissue regeneration. To evaluate the surface charge behavior of the scaffold in acidic and neutral environments, which mimic physiological conditions, the zeta potential of PLCL fibers was measured in a KCl solution across a wide pH range. The titration curve of PLCL+ and PLCL– samples was analyzed in the pH range from 5.5 to 9, see Figure 3e. At lower pH values (5.5), the PLCL+ scaffold exhibited a zeta potential of approximately −18.78 mV, while PLCL– showed a lower potential (−22.96 mV), indicating a difference of 4.18 mV. As the pH increased, the zeta potential values for both samples became progressively more negative. However, PLCL– consistently demonstrated a more pronounced negative charge across the entire range. At pH 7, which closely mimics physiological conditions such as wound fluid, PLCL+ reached a zeta potential of −26.77 mV, compared to −32.40 mV for PLCL– (a difference of 5.64 mV). This difference was still significant at higher pH values. At pH 7.5, the zeta potentials were −29.19 (PLCL+) and −34.77 mV (PLCL−). At pH 9, the difference increased to 8.87 mV (−35.39 mV compared to −44.26 mV, respectively). These results highlight a consistently more negative surface charge for PLCL– samples, particularly in the physiologically relevant pH range, suggesting enhanced ionization or greater surface exposure of negatively charged groups in comparison to PLCL+. Electrospun PCL, PC, and poly­(3-hydroxybutyrate-*co*-3-hydroxyvalerate) (PHBV) fibers with different voltage polarities also demonstrated varying zeta potential according to pH.^31,34,61^

## 3 *In Vitro* Study

### 3.1 Cell Adhesion, Replication, and Proliferation

Adhesion, replication, and proliferation assays provide complementary evaluations of cell behavior in response to electrospun fibrous scaffolds.^68^ In this study, we analyzed MG-63 osteoblast cell adhesion to PLCL+ and PLCL– fibers and glass as a control sample. To evaluate cell adhesion to PLCL scaffolds, we quantified the average cell density 5 h after seeding. A schematic illustrating the individual steps of the experimental procedure is presented in Figure 4a. This analysis was preceded by a PBS wash step to remove nonadherent cells. Figure S4 in the Supporting Information presents representative confocal images used for adhesion quantification. Despite measurable differences in their zeta potential, the PLCL+ and PLCL– scaffolds demonstrated comparable levels of osteoblast adhesion (Figure 4b). These results suggest that surface charge alone may not be the dominant factor affecting early cell attachment to these scaffolds. The reduced number of adherent cells on PLCL fibers after 5 h in comparison to glass can be attributed to the hydrophobic nature of PLCL scaffolds, which limits the adsorption of adhesive proteins from the culture medium during the initial stages of cell adhesion. Furthermore, the 3D topography of the fibers may require a more extended period for cells to establish integrin-mediated adhesion sites. The focal adhesion sites on polymer fibers are smaller and elliptical but exhibit higher protein density than those on the typical glass surface.^69^ In contrast, glass is hydrophilic, promoting rapid protein adsorption and focal adhesion complex formation within the first few hours of culture.

**Figure 4:**
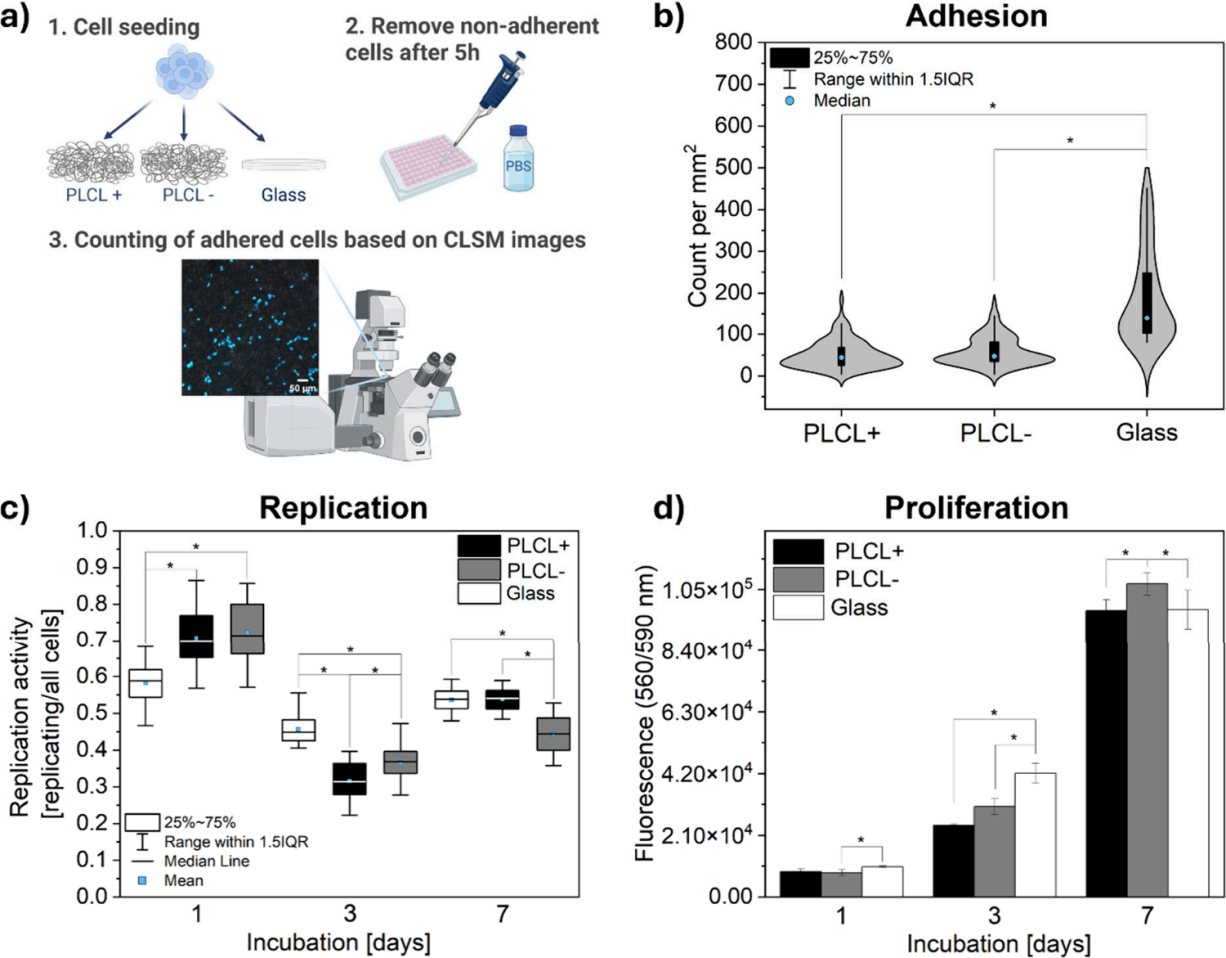
(a) Schematic representation of the cell adhesion analysis protocol (1. Cells were seeded onto the samples and incubated for 5 h; 2. Subsequently, the samples were gently rinsed with PBS to remove nonadherent cells; 3. The number of adhered cells was then estimated based on CLSM imaging, following nuclear staining.), (b) cell adhesion after 5 h of incubation, (c) replication and (d) proliferation assessment after 1, 3, and 7 days of incubation of MG-63 cells on PLCL scaffolds and a glass slide. *Statistical significance calculated with ANOVA followed by Tukey’s post hoc tests, *p* < 0.05.

Given the observation of comparable initial adhesion for both scaffold types, the subsequent investigation focused on determining whether PLCL+ and PLCL– scaffolds differentially influenced cell replication and proliferation. Cell proliferation is a critical step in assessing material biocompatibility and indicates the material’s ability to support tissue regeneration.^9^ Assessment of cell proliferation provides information about how well cells grow and multiply on the material. The replication assay provides a valuable tool for interpreting standard proliferation results and offers additional insight into the dynamics of cell division.^68,70,71^ This method identifies cells actively replicating their DNA by detecting newly synthesized DNA strands within the defined incubation period.^68^ Representative confocal laser scanning microscopy (CLSM) images used to assess DNA replication are shown in Figure S5 in the Supporting Information. Replicative activity was defined as the ratio of replicating nuclei (green) to the total number of nuclei (blue). Figure 4c,d presents the results of cell replication and proliferation on days 1, 3, and 7 of incubation. On days 1 and 3, both PLCL+ and PLCL– scaffolds supported comparable levels of proliferation. However, replication assay data from day 1 revealed that cells cultured on PLCL scaffolds exhibited significantly higher replicative activity compared with the 2D glass substrate. Interestingly, despite this early replication advantage on the 3D fibrous scaffolds, proliferation after 3 days of incubation was higher on the 2D glass surface. This can be attributed to the rapid adhesion and spreading commonly observed on flat, hydrophilic surfaces, which promote faster cell anchorage and cytoskeletal organization.^69^ These factors collectively facilitate the accelerated proliferation. This observation is consistent with our adhesion assay results, which confirmed faster initial cell attachment on glass 5 h after seeding. Notably, after 3 days of incubation, the PLCL scaffolds exhibited higher replication activity than the PLCL+ scaffolds. By day 7, the PLCL– scaffolds demonstrated enhanced proliferation compared with the PLCL+ scaffolds and the glass controls. The reduced proliferation observed on glass at the later time point, despite notably higher replication activity on day 3 compared to fibrous scaffolds, was due to contact inhibition following cell confluence, which restricts further division. In contrast, fibrous PLCL– scaffolds provided a 3D porous architecture that more closely mimics ECM. Although cells initially exhibited slightly reduced metabolic and proliferative activity on fibrous scaffolds, apparently due to the need for topographical adaptation, they subsequently expanded more effectively within the scaffold volume.

### 3.2 Cell Morphology

Morphological assessment was performed to investigate how surface properties modulated by voltage polarity influence cell shape, spreading, and interaction with the fibrous architecture. The morphology of cells on the PLCL scaffolds was assessed using CLSM and SEM. Additionally, as a control, cells were seeded on glass substrates and imaged by confocal microscopy. Imaging was performed after 1, 3, and 7 days of incubation. CLSM images showed that, in both the PLCL+ and PLCL– scaffolds, cells exhibited similar morphology, with no notable differences in cell density (Figure S6 in the Supporting Information). SEM analysis provided further insight into the early stages of cell–scaffold interactions. SEM micrographs confirmed that the 3D structure of the PLCL fibrous scaffold is crucial. The scaffold’s highly porous structure with irregular fiber spacing supported effective cell attachment and spatial adaptation (Figure 5). Cells adhered to individual fibers and spread across and bridged the surrounding gaps. The aligned cytoskeletal organization observed along the fibers suggests that the scaffold architecture supports early integration within the 3D environment by regulating the cell morphology. Notably, as early as day 1 of incubation, cells cultured on PLCL scaffolds exhibited well-developed, elongated actin filaments aligned along the fiber axis, accompanied by visible filopodia anchoring to adjacent fibers as observed via SEM, see Figure 5a,b. These features indicate early cytoskeletal organization and robust cell–scaffold interactions guided by the scaffold’s architecture. A similar phenomenon was reported regarding PCL fibrous scaffolds.^31^ In contrast, cells on PLLA scaffolds displayed predominantly rounded morphologies with minimal actin development at the same time point, suggesting delayed cytoskeletal organization and adaptation.^30^ After 3 days of culture, MG-63 cells exhibited an increasingly elongated morphology, aligning along the fibers on both PLCL+ and PLCL– scaffolds. The cells established multiple contacts with adjacent cells and fibers, indicating progressive adhesion and network formation (Figure 5c,d). After 7 days of incubation, the cells formed a continuous layer covering the scaffold surface. They appeared well-spread and elongated, gradually bridging the interfiber spaces and contributing to partial surface coverage of the fibrous structure (Figure 5e,f).

**Figure 5:**
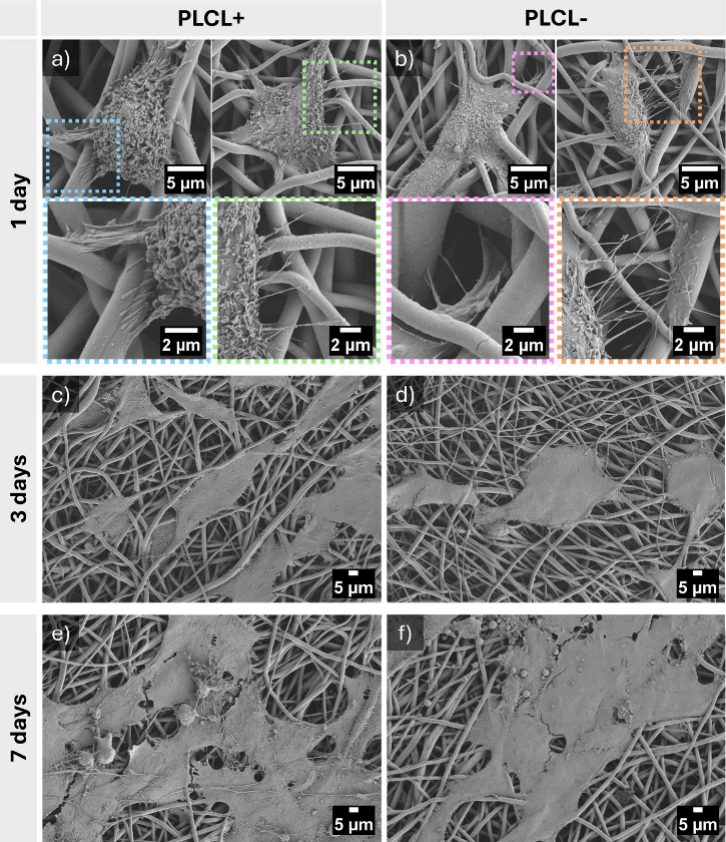
SEM micrographs of MG-63 cells on PLCL+ and PLCL– after various incubation times: (a, b) 1 day (with additional high-magnification micrographs to provide detailed visualization of filopodia formation and cell–fiber interactions), (c, d) 3 days, and (e, f) 7 days.

Moreover, Figure 6a,b highlights 12 representative CLSM slices selected from *z*-stacks acquired for both samples after 7 days of cell culture. Figure 6c illustrates the *z*-stack acquisition and analysis strategy employed to evaluate cell infiltration and integration at various depths within the scaffolds. These images confirm the spatial adaptation of cells along the fibrous architecture and reveal distinct cytoskeletal features including filopodia and lamellipodia. Together, these structures indicate dynamic cell–scaffold interactions and cytoskeletal remodeling in response to the scaffold’s topography.

**Figure 6:**
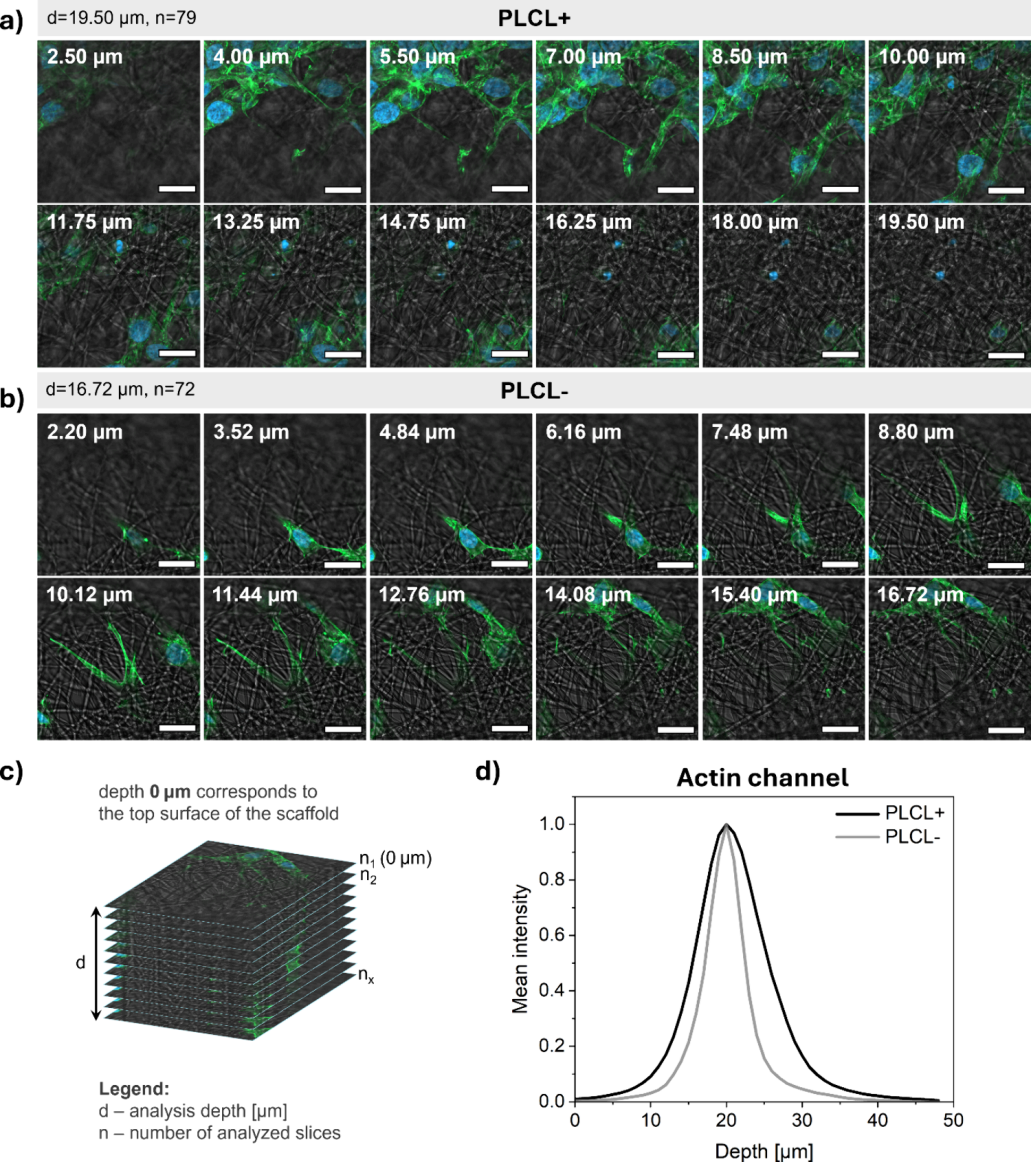
Representative *z*-stack confocal images of MG-63 cells cultured on PLCL scaffolds after 7 days of incubation on (a) PLCL+ and (b) PLCL– (scale bar: 20 μm. Cell nuclei were stained with DAPI (blue), and actin filaments with Alexa Fluor 488 Phalloidin (green)), (c) schematic representation of the *z*-stack acquisition and analysis strategy, (d) normalized intensity profiles of actin fluorescence signal across different imaging depths (*z*-axis) for PLCL+ and PLCL– scaffolds on day 7 of incubation (depth 0 μm corresponds to the top surface of the scaffold).

In addition to *z*-stack imaging, quantitative analysis of fluorescence signal intensity from actin filaments was performed using CLSM *z*-stack images collected over a 320 × 320 μm^2^ field of view with a total *z*-scan depth of 50 μm and a *z*-step of 1 μm. For each optical section, the mean pixel intensity in the actin channel was calculated to determine the depth-dependent distribution of osteoblasts on the PLCL scaffolds after 7 days of incubation. Figure 6d demonstrates the normalized mean intensity profiles of actin fluorescence across the 50 μm *z*-stack depth of the PLCL scaffolds. The sharp, narrow peak centered around a scanning depth of approximately 20 μm corresponds to the layer of cells growing on the scaffold surface. This position indicates the optical plane where the polymer fibers bearing adherent cells were located during imaging. A gradual decay of the signal toward deeper regions (up to 50 μm) reflects a limited yet detectable cellular integration into the fibrous structure. The PLCL+ scaffold exhibited a broader actin intensity profile, with a full width at half-maximum (fwhm) of 10.48 ± 0.15 μm compared to the 5.91 ± 0.13 μm observed in the PLCL– scaffold. A larger fwhm value suggests that cells occupy a thicker interfacial region and penetrate more deeply into the scaffold. This behavior likely results from microstructural differences, such as higher fiber flexibility and porosity in PLCL+, which facilitate improved cell adaptation and infiltration. Consequently, the broader actin distribution highlights the enhanced scaffold performance in promoting three-dimensional cell–scaffold interactions. Additionally, SEM images of cell morphology and distribution (Figure 5e,f) corroborate the quantitative analysis of actin signal intensity across *z*-stack depths on day 7, further emphasizing the distinct cell behaviors observed between PLCL+ and PLCL– scaffolds. Besides, the Movies S1 and S2 in the Supporting Information present 3D *z*-stack reconstructions obtained by CLSM of PLCL scaffolds cultured with cells for 7 days. Specifically, Movie S1 shows PLCL+ scaffolds, while Movie S2 corresponds to PLCL– after incubation for 7 days. The video shows how cells progressively integrate from the scaffold surface, which is defined as a 0 μm imaging depth, through successive focal planes. Furthermore, microscopic observations revealed that cells on PLCL+ scaffolds closely followed the underlying fiber topography, adapting to the 3D architecture by extending across and into the interfiber spaces and, in some regions, partially infiltrating deeper scaffold layers. This behavior indicates a high degree of spatial adaptation of cells to PLCL+ fibers. In contrast, cells on PLCL– scaffolds appeared more flattened and rigidly spread along the fiber surfaces, suggesting more limited interaction with the 3D scaffold architecture.

Although both the PLCL+ and PLCL– scaffolds exhibited comparable average fiber diameters and similar mean pore sizes (10.80 ± 8.21 μm^2^ for the PLCL+ scaffold and 9.60 ± 7.20 μm^2^ for PLCL−), differences in cellular behavior can be attributed to subtle variations in scaffold architecture and surface properties. Notably, the pore fraction was higher for the PLCL+ scaffold (19.26%) than for the PLCL– scaffold (11.39%), promoting more extensive cellular infiltration and cytoskeletal remodeling. In contrast, lower zeta potential measured for the PLCL– fibers may have altered the initial protein adsorption profile and modulated integrin-mediated adhesion, thereby influencing cell spreading.^31,72,73^ Together, these findings suggest that marginal differences in porosity and surface charge affect the interactions of osteoblasts with scaffolds. This could explain the enhanced cell integration and 3D adaptation observed on PLCL+ scaffolds as opposed to the more flattened morphology observed on PLCL– surfaces.

### 3.3 Assessment of Collagen Expression

The ECM provides mechanical strength and regulates essential cellular functions, including adhesion, migration, differentiation, and gene expression, through interactions with specific cell receptors.^74^ Among the ECM components, collagens are the most abundant and structurally dominant proteins, forming fibrillar networks that are critical for the structural integrity of all connective tissues, including bone.^75–77^ Given the essential role of collagen, particularly type I collagen, in maintaining the extracellular framework and promoting osteoblast function, the evaluation of collagen expression is critical for assessing the regenerative potential of fibrous scaffolds. Therefore, in this study, we conducted an analysis of collagen production by MG-63 osteoblast-like cells cultured on PLCL scaffolds. Importantly, only after 14 days, both the PLCL+ and PLCL– scaffolds exhibited well-developed, highly branched collagen networks that formed a 3D structure; see Figure 7a. The collagen networks showed a similar pattern for both PLCL scaffolds.

**Figure 7:**
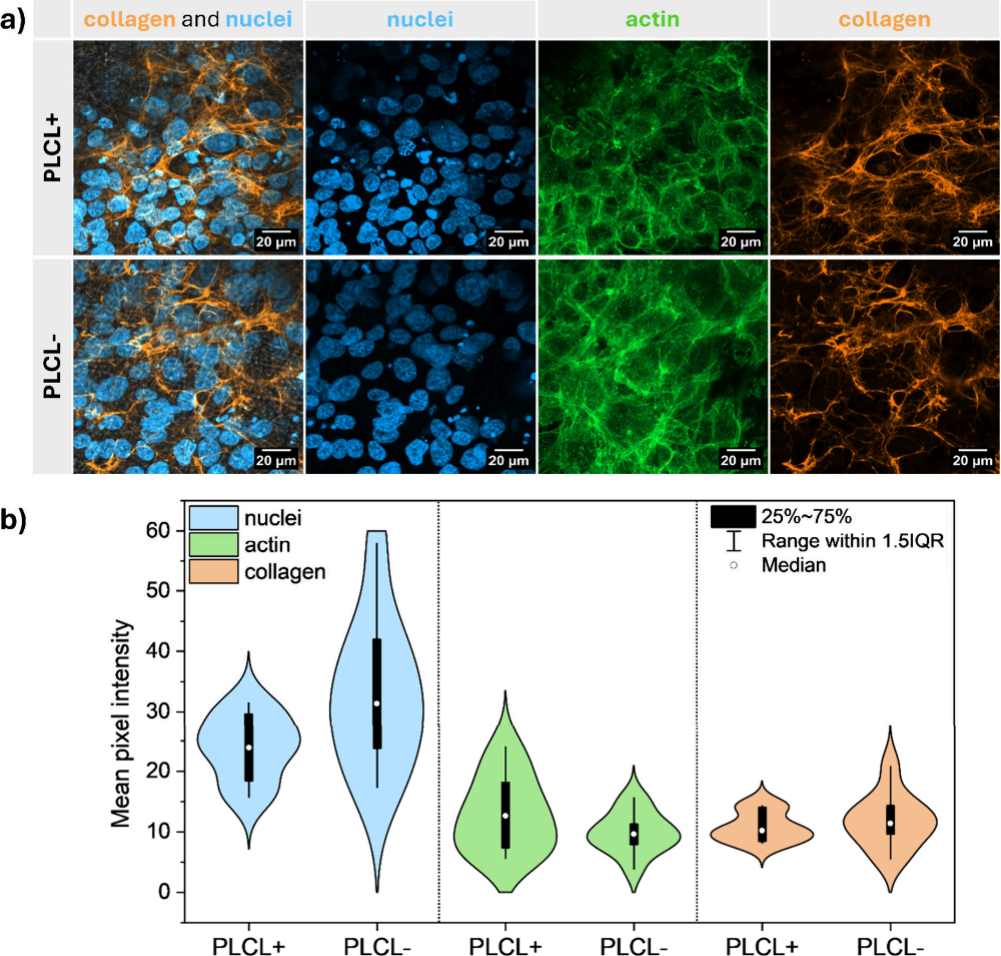
(a) CLSM images of collagen and nuclei after 14 days incubation of MG-63 cells on PLCL+ and PLCL– as well as representative confocal microscopy images of nuclei, actin, and collagen used for the analysis of intensity profiles from the fluorescence signals. CLSM images show collagen (orange), actin filaments (green), and nuclei (blue) stained with Alexa Fluor Plus 555, Alexa Fluor 488 Phalloidin, and DAPI, respectively. (b) Intensity profiles of the fluorescence signals of nuclei, actin, and collagen for the PLCL+ and PLCL– scaffolds on day 14 of incubation.

Additionally, we performed pixel intensity analysis of fluorescence signals corresponding to collagen, actin, and cell nuclei as shown in Figure 7b. To ensure reliable comparison, all imaging parameters were kept consistent across channels (actin, nuclei, collagen). While collagen staining reflects ECM deposition, actin and nuclear signals served as internal references for cytoskeletal organization and cell density, respectively. This multichannel approach allowed us to evaluate collagen expression within the broader context of cellular organization in the scaffold. The analysis confirmed that the comparable collagen expression observed in both PLCL+ and PLCL– scaffolds was not influenced by differences in cell number or spatial distribution but demonstrated that both scaffolds possess an intrinsic capacity to support early ECM formation. These findings demonstrate that, compared to PLLA, PLCL scaffolds not only support but also accelerate ECM development during the early stages of cell culture. Dense collagen structures were reported in PLLA scaffolds after 21 days.^30^ However, PLCL scaffolds exhibited mature 3D collagen networks as early as day 14. The rapid formation of these matrix-like architectures highlights PLCL’s strong potential for bone tissue engineering applications. Interestingly, both PLCL scaffolds supported equally efficient collagen deposition. This finding highlights the potential of electrospun PLCL as a scaffold material for early ECM formation and regenerative support.

## 4 Conclusion

This study comprehensively examined how alternating the electrospinning voltage polarity from positive to negative influences the properties and biological performance of PLCL scaffolds. PLCL is a random copolymer composed of PLLA and PCL units with statistically distributed monomer sequences. This random copolymer architecture of PLCL limits large-scale dipole alignment, thereby attenuating polarity-induced modifications. Scaffolds produced under positive (PLCL+) and negative (PLCL−) polarities exhibited similar fiber morphology, hydrophobicity, thermal behavior, crystallinity, and bulk chemistry. Notably, the pore fraction determined by 2D image analysis was higher for the PLCL+ scaffold (19.26%) than that for the PLCL– scaffold (11.39%). Also, XPS revealed polarity-dependent surface chemical differences, with PLCL– exhibiting increased O=C and O–C bond content. Surface potential measured by KPFM showed minor variation, while zeta potential highlighted more pronounced voltage polarity effects in aqueous conditions at pH 7.5, which was −29.19 mV for PLCL+ and −34.77 mV for PLCL–. These variations influenced cell responses to the PLCL scaffolds. Biological assessments showed that both PLCL+ and PLCL– scaffolds promoted similar levels of early osteoblast adhesion. Replication assays showed that cells on both scaffold types exhibited higher early replicative activity than those on 2D surfaces, reflecting the stimulatory effect of the 3D environment. By day 3, PLCL– scaffolds supported greater replicative activity than PLCL+ scaffolds, and by day 7, they also promoted significantly higher proliferation. These results demonstrate that subtle polarity-induced differences in surface chemistry, when combined with scaffold architecture, can influence cell proliferation dynamics over time.

Morphological assessments of cells via CLSM and SEM confirmed robust cell–scaffold interactions on both scaffolds, characterized by early cytoskeletal organization, aligned actin filaments, and well-developed filopodia anchoring already visible after just 1 day of incubation. This rapid cytoskeletal development highlights the cells’ swift and effective adaptation to the fibrous environment, suggesting favorable interactions with the PLCL scaffold architecture. Notably, cells on PLCL+ scaffolds exhibited deeper spatial infiltration and broader distribution across scaffold depths, likely facilitated by the larger porous area fraction. Conversely, cells on PLCL– scaffolds adopted a more flattened morphology with limited penetration into the scaffold interior, possibly influenced by the more negative surface charge altering protein adsorption and integrin-mediated adhesion dynamics. Importantly, both scaffold types supported strong collagen type I expression after just 14 days of culture, confirming their ability to promote ECM formation and bone tissue regeneration.

We emphasize that even subtle variations in surface chemistry, charge, and microarchitecture can significantly affect osteoblast behavior and 3D integration within fibrous scaffolds. The random copolymer structure of PLCL, while limiting extensive dipole reorientation and surface potential modulation, still allows for fine-tuning of biological responses through control of the electrospinning parameters and scaffold design. Future studies contrasting random and block copolymer architectures will be essential to further elucidate the relationships among monomer sequence, surface responsiveness, and cell–material interactions, ultimately guiding the development of optimized scaffolds for regenerative medicine applications.

## 5 Experimental Section

### 5.1 Materials

Prior to the solution preparation, the poly­(l-lactide-*co*-ε-caprolactone) in a 70/30 molar ratio (PLCL, PURASORB PLC 7015, inherent viscosity midpoint of 1.5 dL/g, Corbion, The Netherlands) was dried by 4 h at *T* = 30 °C in a laboratory drying machine (Pol-Eko, Poland). A polymer solution of 10 wt % concentration was prepared by dissolving PLCL in a mixture of chloroform, acetic acid, and methanol (all solvents Avantor, Poland) in the ratio of 8:1:1 w/w. First, a measured amount of polymer was mixed and dissolved in chloroform alone for 2 h. After this initial dissolution period, the remaining solvents acetic acid and methanol were added to the solution. The mixture was stirred at *T* = 25 °C and 400 rpm using a magnetic stirrer (IKA, Germany).

### 5.2 Electrospinning

The electrospinning process was carried out by using a device equipped with a climate chamber (IME Technologies, The Netherlands). Electrospun random fibers were under either positive (PLCL+) or negative (PLCL−) voltage polarities. The samples were produced by applying a voltage of +15 or −15 kV to the nozzle, with a solution flow rate of 3 mL/h and a distance of 18 cm between the nozzle and the collector, in a climate-controlled chamber at *T* = 25 °C and RH = 40%. A stainless-steel needle (hypodermic injection needle KD Fine 0.8 mm × 40 mm, 21 G × 1 1/2′′ green, KD Medical GmbH Hospital Products, Germany) was used. Fibers were electrospun onto paper on a rotating collector (50 rpm) for 1 h.

### 5.3 Scanning Electron Microscopy (SEM)

The morphology of fibers was analyzed by using a scanning electron microscope (SEM, Merlin Gemini II, ZEISS, Germany). Prior to imaging, all samples were coated with an 8 nm layer of Au by using a rotary pump sputter coater (Q150RS, Quorum Technologies, UK). The operating parameters included an accelerating voltage of 2.5 kV, a current of 110 pA, and a working distance of 4 to 7 mm. The average diameter (*D*_f_) was calculated by analyzing 100 randomly selected fibers from SEM micrographs using ImageJ software (version 1.54g, USA). In the present analysis, only fibers located on the top surface of the electrospun scaffolds were measured, while fibers from lower layers were intentionally excluded to avoid potential errors associated with incomplete focus or overlapping structures. Fiber size distribution histograms were generated using OriginPro software (version 2025 SR1, OriginLab Corporation, USA).

### 5.4 Contact Angle Measurement

The wetting properties of the scaffolds were analyzed by using static advancing contact angle (θ_adv_) measurements. Under controlled conditions (temperature: 23 °C, relative humidity: 40%), 3 μL of deionized water (DI, Spring 5UV purification system; Hydrolab, Poland) was deposited on the surface of the PLCL scaffold on a horizontally positioned sample. The droplet image was captured 3 s after deposition using a camera equipped with a macro lens (EOS 700D, EF-S 60 mm f/2.8 Macro USM, Canon, Japan). The static contact angle was determined by analyzing the images of 10 individual droplets using ImageJ software (version 1.54g, USA) and calculating the standard deviation.

### 5.5 Mechanical Test

To evaluate the mechanical properties of the manufactured fibrous scaffolds, a tensile testing apparatus equipped with a 20 N load cell (Kammrath Weiss GmbH, Germany) was employed. The fibers were uniaxially stretched at a constant rate of 25 μm·s^–1^. Tests were conducted at *T* = 25 °C and RH = 50%. The thickness of the samples was measured by using a stationary thickness gauge (TMG-1-T, Checkline, Germany). Stress–strain curves were generated by using OriginPro software (version 2025 SR1, OriginLab Corporation, USA). To determine the average values for tensile strength (σ_max_), strain at maximum stress (ε_max_), strain at failure (ε_f_), and toughness (*W*), three separate measurements were taken for each sample, and the data were processed using the Integrate function in OriginPro software. Errors are based on the standard deviation.

### 5.6 Thermal Properties (DSC)

Thermal analysis was carried out using differential scanning calorimetry (DSC). Measurement was conducted on a DSC 3 (Mettler Toledo, Switzerland) to determine glass transition temperature (*T*_g_), melting temperature (*T*_m_), and crystallinity (χ_c_). The samples of PLCL pellets, PLCL+, and PLCL– were placed in an Al crucibles, and the measurements were carried out in a dynamic Ar atmosphere (75.0 mL/min). Samples of 9–11 mg were heated from −35 to 220 °C at 10 °C/min. After this first scan, the sample was cooled at 10 °C min^–1^ and reheated for the second scan from −35 to 220 °C at 10 °C min^–1^. Three independent examinations of each sample were performed. *T*_g_ was determined in the first and second scans from the inflection point of the heat flow curve. *T*_m_ and χ_c_ were determined from the first scan. The degree of crystallinity (χ_c_) of the PLCL fibers was calculated using eq 1:

1 $$\chi_{c}=\frac{\Delta H_{m}}{\Delta H_{m}^{{}^{\circ}}\times \left(\mathrm{LA}\right)}\times 100\%$$

where Δ*H*_m_ is enthalpy of melting crystallization (the area under the melting peak). Fernández (2012) confirmed that only lactide units in PLCL 7030 are able to crystallize.^35^ Therefore, Δ*H*_m_^0^ = 106 J·g^–1^ of PLLA) represents the enthalpy of fusion of PLLA crystals with infinite crystal thickness, whereas (LA) denotes the l-lactide fraction in the PLCL copolymer.^78^

### 5.7 Chemical Analysis

Fourier transform infrared spectroscopy (FTIR) was employed to investigate the bulk chemistry and to identify the functional groups in PLCL+ and PLCL– scaffolds using a Nicolet iS5 spectrometer (Thermo Fisher Scientific, USA). The measurements were conducted with the diamond ATR module (iD7 ATR, Diamond) over 64 scans, maintaining a resolution of 4 cm^–1^ and a wavelength range between 400 and 4000 cm^–1^. The FTIR spectra were interpreted by using OMNIC 9 software (version 9.12.928, Thermo Fisher Scientific, USA), and the resulting graphs were prepared by using OriginPro software (version 2025 SR1, OriginLab Corporation, USA).

The surface chemistry of the fibrous scaffold was characterized by using angle-resolved X-ray photoelectron spectroscopy (ARXPS). The preparation of the samples entailed the deposition of fibers onto silicon wafers attached to a rotating collector (2500 rpm) for a duration of 20 min. The ARXPS was carried out in a PHI VersaProbeII Scanning XPS system using monochromatic Al Kα (1486.6 eV) X-rays focused to a 100 μm spot. The photoelectron take off angle was 45°, and the pass energy in the analyzer was set to 46.95 eV (0.1 eV step) to obtain high energy resolution spectra for the C 1s, Si 2p, and O 1s regions. A dual beam charge compensation with 7 eV Ar^+^ ions and 1 eV electrons were used to maintain a constant sample surface potential regardless of the sample conductivity. All XPS spectra were charge referenced to the unfunctionalized, saturated carbon (C–C) C 1s peak at 285.0 eV. The operating pressure in the analytical chamber was less than 2 × 10^–9^ mbar. Deconvolution of spectra was carried out using PHI MultiPak software (v.9.9.3). Spectrum background was subtracted using the Shirley method.

### 5.8 Surface Potential Measurements

Streaming zeta potential analysis was performed using an electrokinetic analyzer (SurPASS 3, Anton Paar, Austria). PLCL scaffolds were placed in a cylindrical cell designed for porous samples. The pH was controlled within the range of 5.5 to 9.0. Titrations were conducted by progressively adding 0.05 M NaOH to a 0.01 M KCl solution. The permeability index during electrolyte flow was approximately 130. Zeta potential measurements were repeated four times for both samples at each pH value. The titration curves are presented as average values with error bars calculated from four measurements.

Atomic force microscopy (AFM) and Kelvin probe force microscopy (KPFM) analyses were performed by using a CoreAFM system (Nanosurf, Switzerland). Conductive HQ:NSC18/Pt tips (MikroMasch, Bulgaria) with a force constant of 2.8 N m^–1^ and a resonance frequency of 75 kHz were used for the KPFM measurements. Topographical data were simultaneously collected during KPFM scanning. For verification and control purposes, measurements from the ITO glass were acquired alongside those from the fibers. The KPFM values presented are averages calculated from 3 separate scans. Data analysis was conducted using Gwyddion software (v2.56, gwyddion.net) and OriginPro (version 2025 SR1, OriginLab Corporation, USA). Measurements were carried out under controlled environmental conditions of 50% relative humidity and 23 °C.

### 5.9 *In Vitro* Studies

The *in vitro* studies were performed on PLCL scaffolds using human osteoblast-like MG-63 cells (Sigma-Aldrich, UK). PLCL+ and PLCL– scaffolds were prepared by cutting them into 15 mm diameter circles and placed into 24-well plates, while a cover glass (Ø 11 mm, Menzel-Glaser, Germany) was used as a control sample for all cell culture tests. All samples were sterilized under UV light for 30 min before the experiment. The culture media used in this research consisted of 86 mL of Dulbecco’s modified Eagle’s medium (DMEM, containing 4.5 g/L glucose, Biological Industries, Israel), 10 mL of fetal bovine serum (FBS, Biological Industries, Israel), 2 mL of penicillin–streptomycin antibiotics (Biological Industries, Israel), 1 mL of l-glutamine solution (Biological Industries, Israel), and 1 mL of nonessential amino acids (100× solution, Sigma-Aldrich, USA). The samples were incubated at 37 °C in an atmosphere of 95% humidity and 5% CO_2_ using an incubator (Memmert GmbH + Co. KG, Inc. 108med, Schwabach, Germany).

### 5.10 Cell Adhesion

The assessment of cell adhesion on the PLCL scaffolds and glass control samples was conducted after 5 h of incubation. Cells were seeded at a density of 1 × 10^5^ cells per 1 mL of culture media. After the required time from cell seeding, samples were washed in phosphate-buffered saline (PBS, Biomed Lublin, Poland) two times to remove unattached cells. In the next step, scaffolds were fixed with 4% paraformaldehyde for 15 min and rinsed with PBS. For nuclear staining, 4′,6-diamidino-2-phenylindole dye (DAPI, Sigma-Aldrich, UK) incubation was performed for 15 min. After this step, samples were rinsed with PBS. Cell adhesion was assessed using confocal laser scanning microscopy (CLSM, Zeiss LSM 900, Germany). For each sample, 50 representative fields of view were imaged under identical acquisition settings. Cell quantification was performed with CellProfiler 4.2.6 (Broad Institute, USA) software.

### 5.11 Cell Replication

To assess the ability of cells to replicate, cells were grown on PLCL+ and PLCL– scaffolds as well as on a glass slide for use as a control. The assessment was conducted after 1, 3, and 7 days of culture. Initially, the samples were labeled with 10 μM 5-ethynyl-2′-deoxyuridine (EdU) for 1 h. After this step, the samples were washed with PBS and fixed with 4% paraformaldehyde for 15 min. The samples were then washed twice in each well with 3% bovine serum albumin (BSA) (Sigma-Aldrich, USA) in PBS, followed by permeabilization with 0.5% Triton X-100 (Sigma-Aldrich, USA) for 20 min. After removal of the permeabilization buffer, the samples were washed twice in each well with 3% BSA. The incorporated EdU was detected using the Click-iT EdU AF488 Imaging Kit (Invitrogen/Molecular Probes, USA). For nuclear staining, cells were incubated with DAPI (Millipore) for 15 min. Fixed samples were imaged using a Zeiss LSM 900 CLSM (Carl Zeiss Microscopy GmbH) equipped with a Plan-Apochromat 20×/0.8 M27 objective. Excitation was performed with 405 and 488 nm laser lines, and fluorescence emission was collected at 410–500 nm for DAPI and 500–700 nm for Alexa Fluor 488 bound to the incorporated EdU. Over 50 images were captured for each sample from different fields of view. The recording of the images involved two channels: the green channel for detection of the signal from the Alexa 488-labeled EdU precursor incorporated into replicating cells and the blue channel for DAPI-labeled nuclei. Nuclei detection in both channels was performed using CellProfiler 4.2.6 software (Broad Institute, USA). The average density of replicated and total cells per mm^2^ was quantified from at least 40 random fields.

### 5.12 Cell Viability

Cell proliferation was determined after 1, 3, and 7 days of incubation using the CellTiter-Blue Assay (GloMax Discover Plate Reader, Promega, USA). Cells were seeded at a density of 2 × 10^4^ cells per 1 mL of culture media. Before measurement, samples containing attached cells (PLCL+, PLCL–, and glass) were transferred to a new plate to ensure that the analysis focused exclusively on cells that adhered to and proliferated on the surface of the tested scaffolds. Subsequently, 400 μL of fresh medium and 80 μL of CellTiter-Blue reagent (Promega, USA) were mixed and added to the samples, which were then incubated for 4 h at 37 °C. After incubation, 100 μL of the reagent was transferred in triplicate to a 96-well plate, and fluorescence was measured at 560/590 nm using the GloMax Discover System (Promega, USA) microplate reader. For the cell culture study, each sample type was tested in duplicate. Statistical analysis was performed using OriginPro software (version 2025 SR1, OriginLab Corporation, USA). Statistical analysis of cell adhesion, replication, and proliferation was performed using one-way ANOVA followed by Tukey’s post hoc test in OriginPro. Differences were considered statistically significant for *p* < 0.05.

### 5.13 Cell Imaging (CLSM and SEM)

The cells were seeded on each PLCL+ and PLCL– sample and glass and cultured for 1, 3, and 7 days. Subsequent to each designated time point, the samples were fixed with 4% paraformaldehyde (Sigma-Aldrich, UK) for 15 min. Following this fixation step, the samples were thoroughly rinsed with PBS. Subsequently, the samples were incubated in 0.1% Triton X-100 (Sigma-Aldrich, UK) for 10 min, followed by rinsing in PBS. The blocking step involved incubation in 3% BSA in PBS for a duration of 60 min. The actin cytoskeleton was stained with Alexa Fluor 488 phalloidin (Thermo Fisher Scientific, USA) for 1 h at 23 °C, and nuclei were counterstained with 4′,6-diamidino-2-phenylindole (DAPI; Sigma-Aldrich, UK) for 15 min. Images were acquired using a Zeiss LSM 900 CLSM (Carl Zeiss Microscopy GmbH) with objectives from 10× to 40×. Excitation was achieved with laser lines at 405 and 488 nm for DAPI and Alexa Fluor 488, respectively.

SEM imaging of cells on PLCL scaffolds was conducted after 1, 3, and 7 days of culture. The cells were fixed with 2.5% glutaraldehyde (Sigma-Aldrich, UK) for 1 h at 25 °C. Subsequently, the samples were rinsed three times with PBS and dehydrated through a graded ethanol series (Avantor, Poland) with increasing concentrations (30%, 50%, 80%, 90%, and 100%). The scaffolds were incubated in each ethanol solution for 7 min with the 100% ethanol step repeated twice. Following dehydration, the samples were incubated in hexamethyldisilazane (HDMS, Sigma-Aldrich, UK) until complete evaporation. Next, the scaffolds were placed on specialized Al tables and subjected to a gold sputtering process using a Quorum Q150AS sputtering machine. The samples were coated with an 8 nm layer of Au in a vacuum environment with a current of 20 mA. After sputtering, the samples were imaged by using the same SEM parameters, and the morphology of the fibers was examined.

### 5.14 Collagen Staining

The immunofluorescence staining of extracellular collagen was conducted on days 7 and 14 following the seeding of cells onto PLCL+ and PLCL– fibers. The cells were incubated at 37 °C for 1 h with a monoclonal anti-collagen type I antibody (C2456, Merck, USA), washed with 5% FBS in PBS, and subsequently fixed with 4% formaldehyde in PBS for 15 min at room temperature (23 °C) followed by two PBS rinses. Permeabilization was carried out with 0.1% Triton X-100 in PBS for 10 min at 23 °C and nonspecific binding was blocked with 3% BSA in PBS for 1 h. Next, the cells were incubated with an Alexa Fluor Plus 555-conjugated anti-mouse secondary antibody (A32727, Thermo Fisher, USA) for 1 h. Actin filaments were visualized by incubating the cells with Alexa Fluor 488 Phalloidin (Thermo Fisher, USA) for 1 h at 23 °C, followed by three PBS washes. Nuclear DNA was counterstained with DAPI (Sigma-Aldrich, UK) for 10 min. After staining, the samples underwent three additional PBS washes, each lasting 15 min. The same laser settings were applied for focal adhesion imaging using a confocal microscope.

For the quantitative analysis of collagen, actin, and nuclear staining, images were acquired using identical acquisition parameters, including excitation laser intensity, detector gain, emission detection ranges, and pixel size. Images covered an area of 638 μm with a pixel size of 125 nm. For each sample, 10 randomly selected fields of view were imaged. Autofocus was used to identify the Z-plane with the highest signal intensity, and separate channels were recorded for collagen, actin, and nuclei. Quantification was performed by calculating the mean pixel intensity for each marker. To account for variations in cell density and staining efficiency, the collagen signal was normalized to reference signals from actin and nuclear staining obtained from the same fields of view.

## Supplementary Material







## Data Availability

The data that support the findings of this study are available from the corresponding author upon reasonable request and are included in the Supporting Information.

## Notes

Pore size distribution plot of PLCL scaffolds; representative images of water droplets for static contact angles measurements for PLCL scaffolds; stress–strain curves for PLCL+ and PLCL–; summary of FTIR results for PLCL+ and PLCL– scaffolds and PLCL pellets; schematic explanation of polymer chain reorientation in electrospinning after applying a positive and negative voltage polarity; surface composition (atomic %) determined by fitting ARXPS data for PLCL+ and PLCL– samples; confocal microscopy images of MG-63 cells cultured on PLCL+, PLCL–, and glass for 1, 3, and 7 days; representative confocal microscopy images of MG-63 cells used for adhesion analysis on PLCL+ and PLCL– and glass; representative CLSM micrographs of MG-63 cells on PLCL+, PLCL–, and glass slides used for analysis of cell replication (PDF)
Movie S1. 3D CLSM z-stack of PLCL+ scaffold cultured with MG-63 cells for 7 days (PDF)
Movie S2. 3D CLSM z-stack of PLCL– scaffold cultured with MG-63 cells for 7 days (PDF)
