# Supplementary material for: Modulating Surface Properties and Osteoblast Responses in Bone Regeneration via Positive and Negative Charges during Electrospinning of Poly(l‑lactide-co-ε-caprolactone) (PLCL) Scaffolds
Source: ACS Biomater Sci Eng. 2025 Nov 27;12(1):543–58. doi: 10.1021/acsbiomaterials.5c01568 (PMC12801196; doi:10.1021/acsbiomaterials.5c01568)
Supplement: Supplementary file 1 [file ab5c01568_si_001.pdf]

Supporting Information

Modulating surface properties and osteoblast responses in bone regeneration via positive and negative charges during electrospinning of PLCL scaffolds

Katarzyna Marszalik [a], *Martyna Polak* [a], *Krzysztof Berniak* [a], *Joanna Knapczyk-Korczak* [a], *Piotr K. Szewczyk* [a], *Mateusz M. Marzec* [b], *Urszula Stachewicz* [a*]

[a] Faculty of Metals Engineering and Industrial Computer Science, AGH University of Krakow, Al. A. Mickiewicza 30, Krakow 30-059, Poland

[b] Academic Centre for Materials and Nanotechnology, AGH University of Krakow, Al. A. Mickiewicza 30, Krakow 30-059, Poland

Email: ustachew@agh.edu.pl

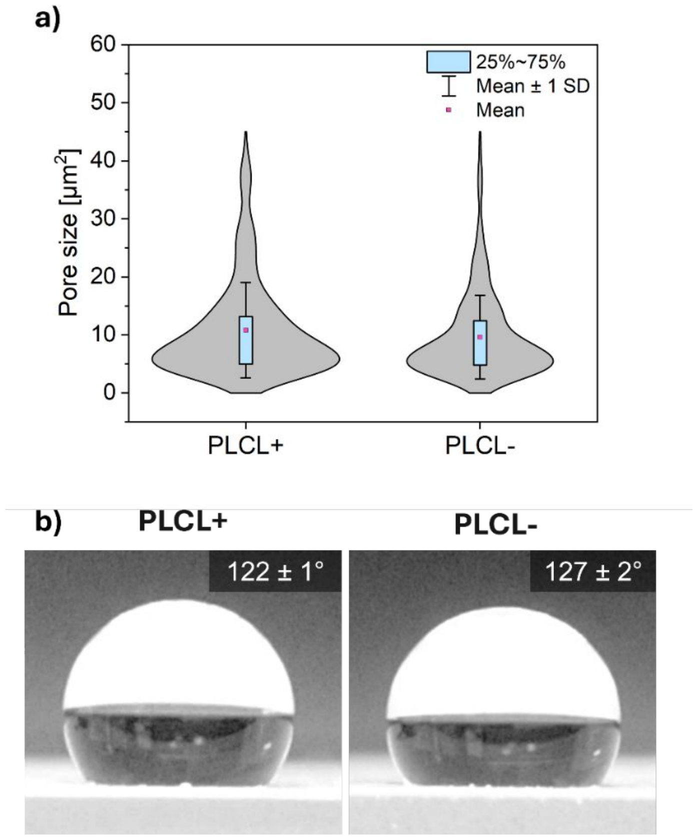

Figure S1. (a) pore size distribution plot determined by 2D analysis of SEM micrographs of PLCL+ and PLCL- fibrous scaffolds, (b) representative images of water droplets for static contact angles measurements for PLCL+ and PLCL- electrospun fibers.

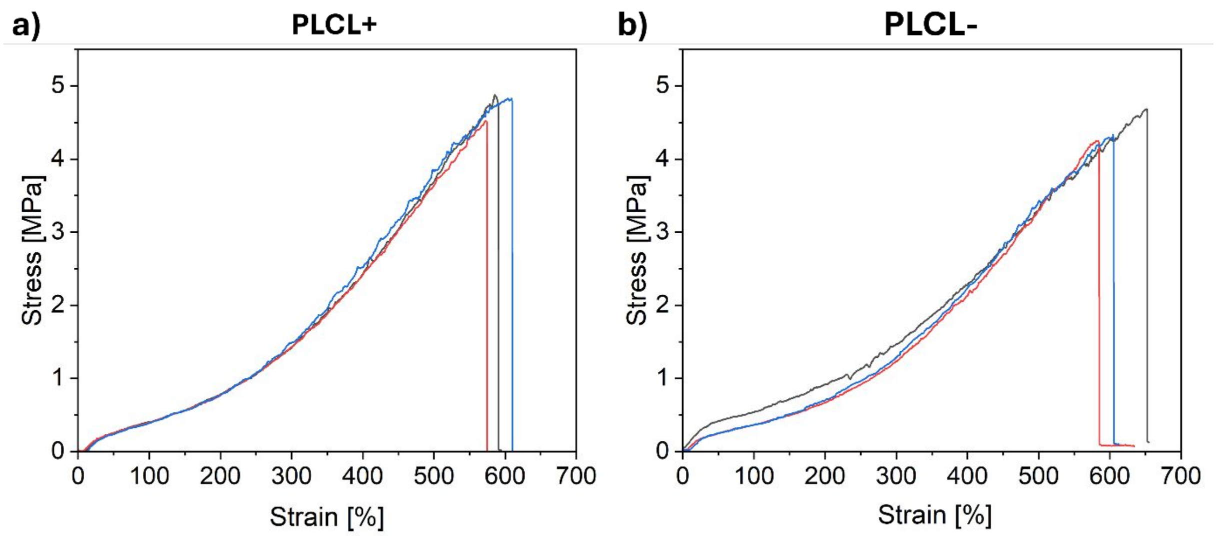

Figure S2. Stress-strain curves for (a) PLCL+, (b) PLCL-.

Table S1. Summary of FTIR results for PLCL+ and PLCL- scaffolds and PLCL pellets, showing characteristic absorption peaks (wavenumber in cm^{-1}) and their corresponding chemical bond assignments.

Wavenumber (cm^{-1})			Corresponding bond
PLCL pellets	PLCL+	PLCL-	
2944	2944	2943	C-H stretching[1]
1747	1746	1746	C=O stretching[2,3]
1454	1452	1452	$-CH_3$ bending[3]
1359	1359	1359	
1181	1181	1181	C-O stretching[2]
1 129	1128	1128	
1 084	1084	1084	
1 043	1043	1043	
871	869	869	C-COO stretching[4]

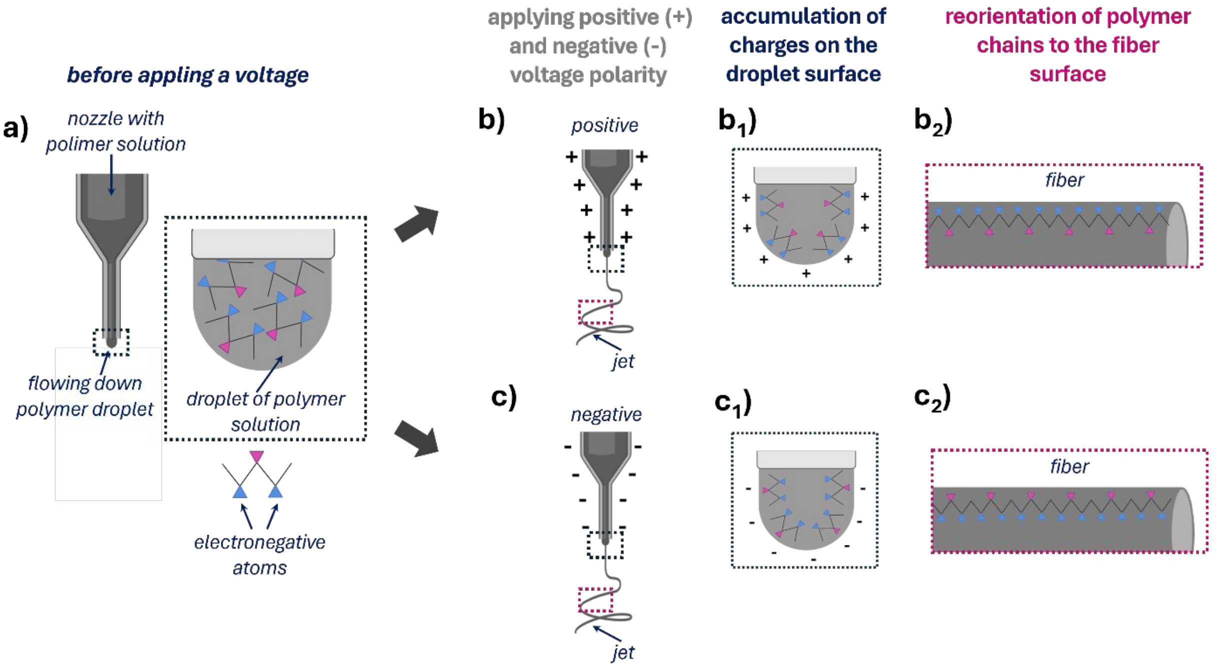

Figure S3. Schematic explanation of polymer chain reorientation in electrospinning after applying a positive and negative voltage polarity: (a) flowing down of a polymer droplet at the end of the nozzle before applying voltage polarity. Accumulation of charges on the droplet

surface after applying (b, b_1) positive and (c, c_1) negative voltage polarity, (b_2, c_2) scheme of fibers demonstrating reorientation of polymer chains on the surface of fibers.[5]

Table S2. Surface composition (atomic %) determined by fitting ARXPS data for PLCL+ and PLCL- samples at 45°, corresponding to 5.7 nm measurement depth.

		C			O		Si	
Binding energy [eV]		285.0	286.7	289.0	532.1	533.5	99.0	102.9
Sample	Measurement depth [nm]	C-C	C-O	O-C=O	O=C	O-C	Si^0	SiO_2
PLCL+	5.7	36.1	16.8	16.0	13.6	16.6	0.9	0.2
PLCL-		31.3	16.2	16.3	15.4	17.8	2.3	0.6

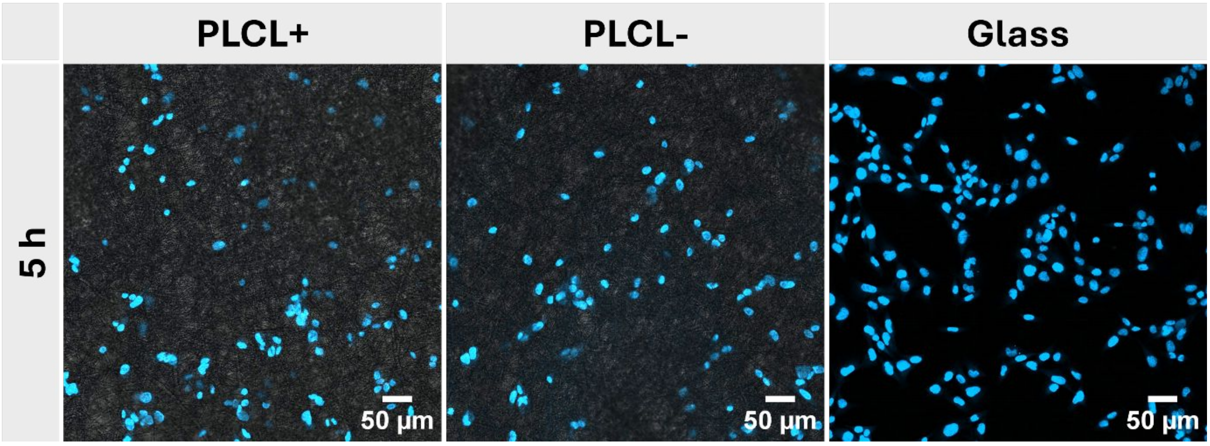

Figure S4. Representative confocal microscopy images of MG-63 cells used for adhesion analysis after 5 h on PLCL+ and PLCL- fibrous scaffolds, as well as on the control surface (glass). Nuclei are stained with DAPI (blue).

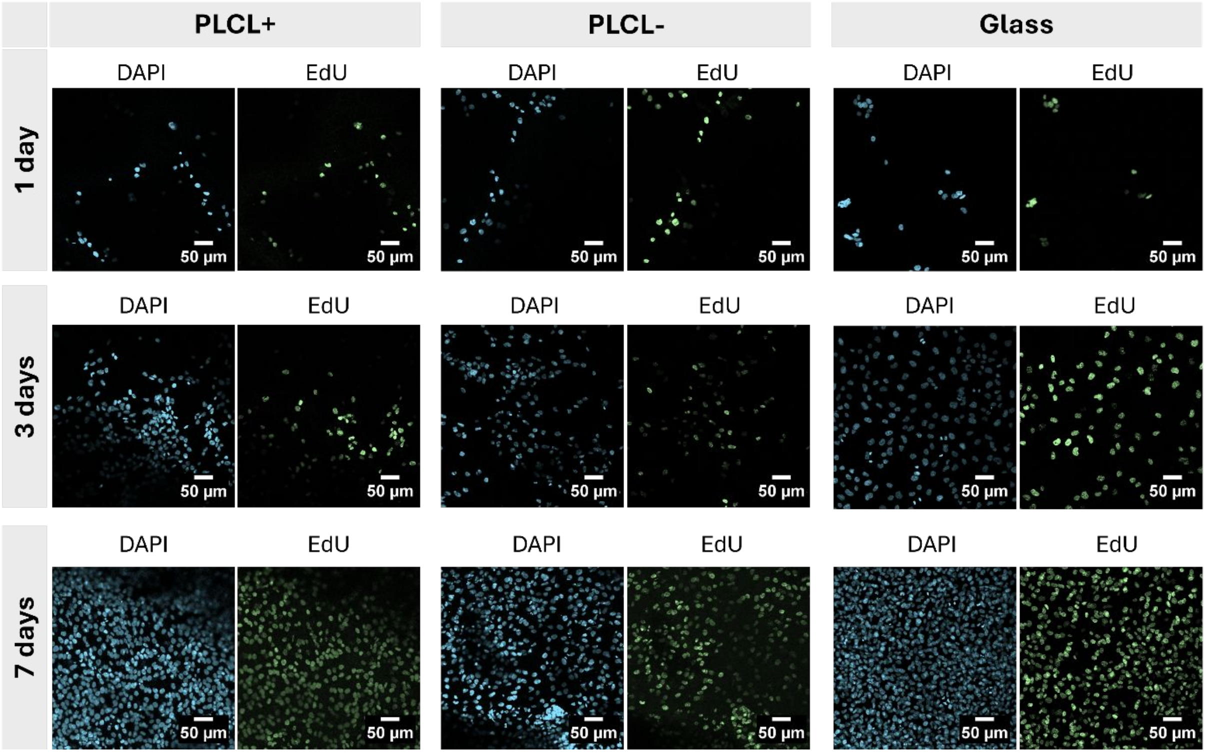

Figure S5. Representative CLSM micrographs of MG-63 cells on PLCL+, PLCL-, and glass slides used for analysis of cell replication. Nuclei are stained with DAPI (blue), and incorporated EdU during DNA replication is stained with Alexa Fluor 488 (green).

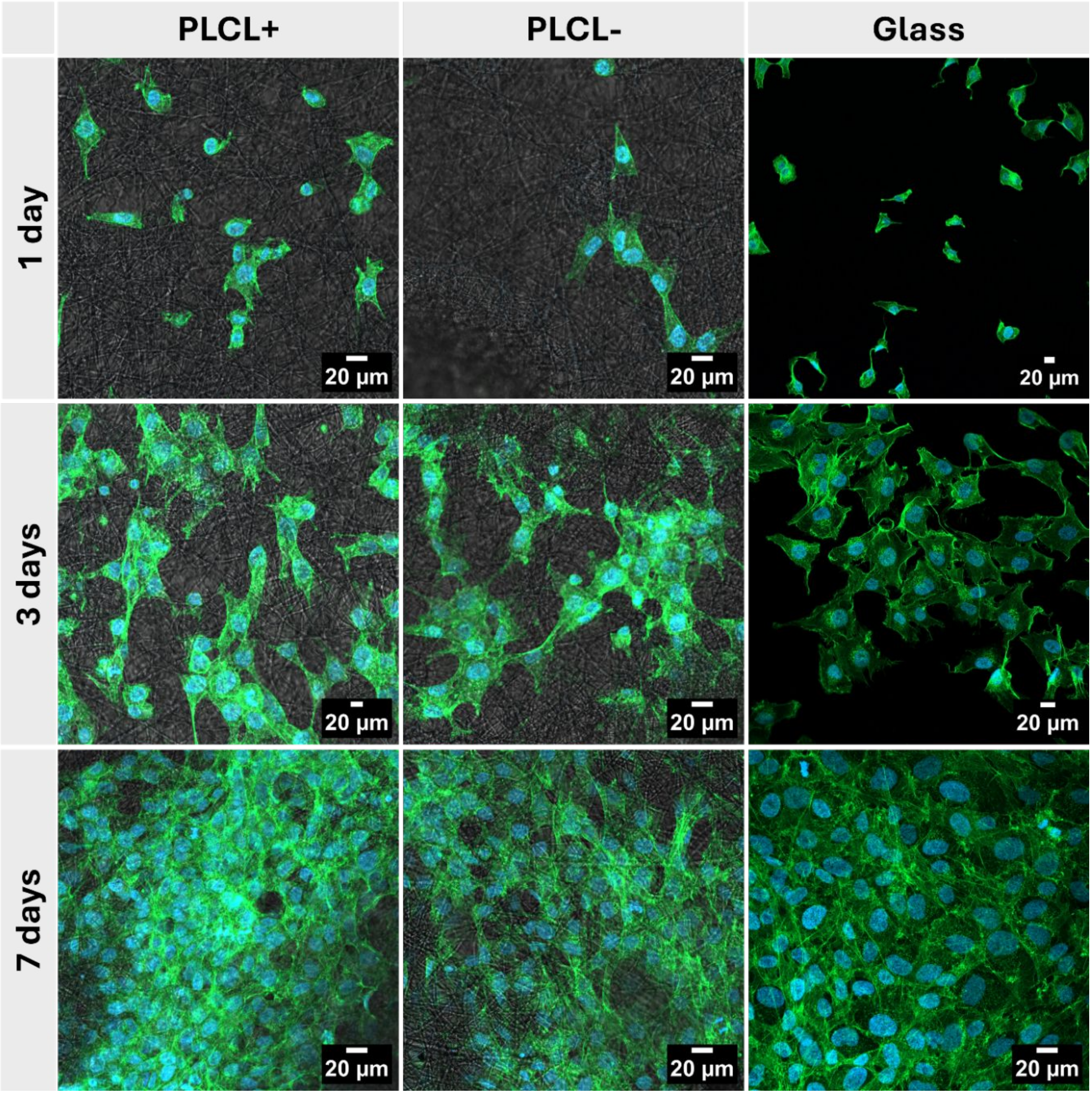

Figure S6. Confocal microscopy images of MG-63 cells cultured on PLCL+, PLCL- and glass for 1, 3, and 7 days, showing cytoskeletal organization and cell spreading. Nuclei are stained with DAPI (blue), and actin filaments with Alexa Fluor 488 Phalloidin (green).

REFERENCES

(1) Inthanon, K.; Daranarong, D.; Techaikool, P.; Punyodom, W.; Khaniyao, V.; Bernstein, A. M.; Wongkham, W. Biocompatibility Assessment of PLCL-Sericin Copolymer Membranes Using Wharton's Jelly Mesenchymal Stem Cells. *Stem Cells Int* **2016**, *2016* (1). https://doi.org/10.1155/2016/5309484.

(2) Garkhal, K.; Verma, S.; Jonnalagadda, S.; Kumar, N. Fast Degradable Poly(L-Lactide-Co-e-Caprolactone) Microspheres for Tissue Engineering: Synthesis, Characterization,

and Degradation Behavior. *J Polym Sci A Polym Chem* **2007**, *45* (13), 2755–2764. https://doi.org/10.1002/pola.22031.

(3) Larrañaga, A.; Guay-Bégin, A.-A.; Chevallier, P.; Sabbatier, G.; Fernández, J.; Laroche, G.; Sarasua, J.-R. Grafting of a Model Protein on Lactide and Caprolactone Based Biodegradable Films for Biomedical Applications. *Biomatter* **2014**, *4* (1), e27979. https://doi.org/10.4161/biom.27979.

(4) Orhan, H.; Yilmaz, B. In Vitro Properties of Electrospun Composite Fibers Containing Boric Acid and Enhanced with Epidermal Growth Factor for Wound Dressing Applications. *Fibers and Polymers* **2024**, *25* (2), 485–500. https://doi.org/10.1007/s12221-023-00454-8.

(5) Ura, D. P.; Stachewicz, U. The Significance of Electrical Polarity in Electrospinning: A Nanoscale Approach for the Enhancement of the Polymer Fibers' Properties. *Macromol Mater Eng* **2022**, *307* (5). https://doi.org/10.1002/mame.202100843.
